# Early COVID-19 Pandemic Preparedness: Informing Public Health Interventions and Hospital Capacity Planning Through Participatory Hybrid Simulation Modeling

**DOI:** 10.3390/ijerph22010039

**Published:** 2024-12-30

**Authors:** Yuan Tian, Jenny Basran, Wade McDonald, Nathaniel D. Osgood

**Affiliations:** 1 Department of Computer Science, University of Saskatchewan, Saskatoon, SK S7N 5C9, Canada; gwm762@mail.usask.ca (W.M.); osgood@cs.usask.ca (N.D.O.); 2 Saskatchewan Health Authority, Saskatoon, SK S7K 0M7, Canada; 3 College of Medicine, University of Saskatchewan, Saskatoon, SK S7N 5E5, Canada

**Keywords:** COVID-19, pandemic preparedness, hybrid simulation, participatory modeling, contact tracing, hospital capacity planning, agent-based modeling, discrete-event simulation, system dynamics

## Abstract

We engaged with health sector stakeholders and public health professionals within the health system through a participatory modeling approach to support policy-making in the early COVID-19 pandemic in Saskatchewan, Canada. The objective was to use simulation modeling to guide the implementation of public health measures and short-term hospital capacity planning to mitigate the disease burden from March to June 2020. We developed a hybrid simulation model combining System Dynamics (SD), discrete-event simulation (DES), and agent-based modeling (ABM). SD models the population-level transmission of COVID-19, ABM simulates individual-level disease progression and contact tracing intervention, and DES captures COVID-19-related hospital patient flow. We examined the impact of mixed mitigation strategies—physical distancing, testing, conventional and digital contact tracing—on COVID-19 transmission and hospital capacity for a worst-case scenario. Modeling results showed that enhanced contact tracing with mass testing in the early pandemic could significantly reduce transmission, mortality, and the peak census of hospital beds and intensive care beds. Using a participatory modeling approach, we not only directly informed policy-making on contact tracing interventions and hospital surge capacity planning for COVID-19 but also helped validate the effectiveness of the interventions adopted by the provincial government. We conclude with a discussion on lessons learned and the novelty of our hybrid approach.

## 1. Introduction

The COVID-19 pandemic has caused over five million deaths and has placed unprecedented pressure on healthcare systems worldwide. Especially early in the pandemic, decision-makers were compelled to make rapid decisions to mitigate COVID-19 transmission and prevent hospital capacity crises in the face of limited empirical scientific evidence and a lack of effective treatments. To support their decision-making process, many governments used mathematical models along with other epidemiological methods to monitor the epidemic trend and assess the potential impact of various mitigation interventions [1,2]. An essential component of early pandemic preparedness involved estimating the need for hospital beds and healthcare resources under various intervention measures. In Canada, provinces and territories, each with their own authority over health, have adopted different public health strategies in response to the pandemic, tailored to their unique demographic, geographical, and epidemiological characteristics [3].

Saskatchewan was particularly successful in flattening the epidemiologic curve in the first wave of the pandemic until June 2020 due to its swift response and implementation of various public health measures [3]. In the earliest stages of the COVID-19 pandemic in March 2020, when no effective treatments or vaccines were available, the only options available for reducing COVID-19 transmission and easing the burden on the hospital system were classical non-pharmaceutical public health interventions, such as physical distancing, testing, case isolation, quarantine, and contact tracing. During this initial phase of the pandemic, public health measures in Saskatchewan were informed by a participatory hybrid simulation approach.

This study has two primary objectives: (1) to describe the participatory modeling processes used to engage stakeholders and ensure model buy-in, which directly resulted in the use of models for policy-making and pandemic response under a government mandate; and (2) to develop a novel hybrid simulation approach to model COVID-19 transmission and hospital resource needs, and to inform public health policies and hospital capacity planning in Saskatchewan during the early phase of the pandemic from March to June 2020. The significance of this study lies in the dual focus on both the modeling processes and the simulation model. The participatory modeling processes ensured the model’s relevance, credibility, and direct impact on decision-making; while the hybrid simulation approach allowed for representation of different aspects of the problem and addressing different levels of practical decision needs by combining multiple simulation methods. Although simulation modeling has been widely used in infectious disease research [1,2,4,5,6,7], the actual use of simulation models in decision-making for health policy in the realm of infectious disease remains low, and is rarely reported or operationalized [1,4,8,9,10,11,12]. Using a participatory hybrid simulation approach, our study seeks to bridge this gap by engaging stakeholders throughout the modeling processes, which in turn improves the application of simulation findings in real-world decision-making contexts.

This paper is organized as follows: Section 2.1 provides a brief review of participatory modeling approaches and explains the participatory modeling approach used in this study. Section 2.2 describes the implemented participatory modeling processes. Section 2.3 details the structure of the hybrid COVID-19 simulation model. Model validation and intervention scenarios are presented in Section 2.4 and Section 2.5, respectively. We assessed the likely effects of various combined scenarios of physical distancing, testing, conventional contact tracing, and digital contact tracing under stakeholders’ guidance. Section 3 presents the simulation results. Specifically, Section 3.3 details how the model findings have influenced decision-making and policy responses. Finally, we discuss the challenges encountered and highlight key lessons for future pandemic preparedness in Section 4.

## 2. Materials and Methods

### 2.1. Participatory Modeling to the COVID-19 Pandemic

#### 2.1.1. Our Approach to Participatory Modeling: Purpose and Stakeholder Roles

Participatory modeling approaches refer to a diverse set of methods designed to engage stakeholders in the modeling processes to generate, translate, and improve the use of knowledge [13,14,15,16,17,18,19,20,21,22]. They have been developed and applied in various fields, such as environmental planning [15,16], water management [13,14], and health service research [17,18,19,20,21]. They can be integrated with various simulation modeling methods such as System Dynamics modeling [20,23,24] and discrete-event simulation [25,26,27,28]. The purpose of participatory modeling processes can vary depending on the specific approach. Though participatory modeling approaches have been mostly used as a tool to facilitate shared or community-based learning, they could also be used to inform policy-making, support public health policy developments, and mobilize actions [13,17,18,21,22,29,30,31]. Depending on the intended purpose, the categories of stakeholders to be considered for inclusion and the stages of the modeling process with stakeholder engagement may vary [15,25,32,33].

The participatory modeling processes used in this study were adapted from a previously developed four-stage participatory approach used for policy-making within the Saskatchewan context, and the details of this participatory modeling approach have been described elsewhere [34]. The most notable characteristic of our participatory modeling approach is the active engagement of knowledge users in the modeling processes. Knowledge users are stakeholders who can identify a problem, are positioned to use the model findings, and have the authority to implement the research recommendations [35,36]. Knowledge users play critical roles in co-defining the problem and guiding scenario analyses in the modeling processes. This explicit focus aligns with the main purpose of our participatory modeling approach—using simulation models as decision support tools to generate actionable insights that directly support planning and policy decisions. In contrast, the involvement of knowledge users is often not explicitly stated in traditional participatory modeling approaches or community-based participatory approaches [35]. Our participatory modeling approach also engaged other stakeholders, such as epidemiologists and domain experts, who played key roles in the model parameterization and model validation stages.

#### 2.1.2. Study Context and Background

Participatory modeling processes allow the joint creation of a model with modelers, stakeholders, experts and patients to represent a shared understanding of the problem to build consensus or inform decision-making [13,17,18,21,22,30,31,34]. Notably, prior to the COVID-19 pandemic, the Saskatchewan Health Authority and Ministry of Health had already used participatory modeling approach to make informed decisions in areas such as reducing emergency department (ED) wait times [34,37], planning a new ED, and projecting acute care bed needs for a new hospital. These earlier efforts demonstrated the value of the participatory modeling approach and fostered trust in the simulation methods and in the expertise of the modeling team among stakeholders [34]. Therefore, when the first positive COVID-19 case was detected on 12 March 2020, public health officials and stakeholders sought technical support to understand the epidemiological spread of COVID-19 infection and explore potential mitigation measures. We quickly formed a modeling support team leveraging the trust and collaborative relationships established through earlier participatory modeling work. The modeling team was later seconded to the Saskatchewan Health Authority. The modeling support team conducted participatory modeling processes with public health officials, stakeholders, and epidemiologists to improve the translation of simulation model results into informed decision-making.

### 2.2. Participatory Modeling Processes

We adapted our participatory modeling approach from previous work to address the needs for rapid decision-making in Saskatchewan for the COVID-19 pandemic [34]. Key activities in the earlier participatory modeling process [34]—establishing initial buy-in via proof-of-concept modeling, assembling the modeling team, problem conceptualization, model implementation, model validation, and model use—were simplified and re-used to guide the effort.

1. *Establish initial buy-in via proof-of-concept modeling*: We started developing proof-of-concept epidemiological models of COVID-19 transmission in late January 2020 to simulate the outbreak in China. The first version of the hybrid model presented in this study was completed in early February, before any COVID-19 cases were detected in Canada. This initial work was carried out by the academic modeling team led by N.O. [2]. The early work allowed us to quickly adapt the model to the Canadian context as the pandemic progressed. In addition, our team’s prior success in using participatory modeling for healthcare policy decisions in Saskatchewan [34] had built a solid foundation for collaboration, as many stakeholders were already familiar with dynamic modeling approaches and had worked with the modelers before. These two factors enabled us to act quickly. By 16 March 2020—just four days after the first confirmed COVID-19 case in Saskatchewan—we delivered our first presentation to stakeholders in the Saskatchewan Health Authority. We introduced the modeling approaches and presented early findings from the proof-of-concept COVID-19 modeling work. This meeting was crucial for gaining initial buy-in for using simulation modeling to guide rapid pandemic response. Drawing from experience in earlier participatory modeling work, we presented the modeling approaches in a less technical way and explained how these models could be used as a tool to simulate “what-if” intervention scenarios and make projections on epidemic curves under different assumptions to support decision-making. We clarified that the models were developed for making projections but not predictions. This distinction helped stakeholders understand the scope and purpose of these simulation models and avoided potential misinterpretation or misuse of the model results. This is crucial in the early stages when there were so many unknowns about COVID-19 transmission. The presentation included projections of epidemic curves under various intervention scenarios, including preliminary results of how enhanced contact tracing could help flatten the curve using an earlier version of the hybrid model presented in this study. Given that Saskatchewan was still in a very early stage of the COVID-19 pandemic, we highlighted both challenges and immediate actions that could be taken to mitigate the transmission. The initial modeling work drew significant interest among the stakeholders, leading to a follow-up presentation to the senior executive leadership team in Saskatchewan Health Authority on 19 March 2020.
2. *Assembling the modeling support team*: The quick response and use of proof-of-concept COVID-19 models helped secure buy-in with the stakeholders. By the end of March 2020, the academic modeling team was seconded to the Saskatchewan Health Authority with the mandate to provide modeling support for the province’s pandemic response. The team was co-led by J.B. and N.O., with infectious disease modelers (including Y.T. and W.M.), data analysts, and additional analytical support from Saskatchewan Health Authority and Ministry of Health. Notably, three members of the team (Y.T., N.O., and J.B.) were also key members in earlier participatory modeling work [34], who had worked closely with stakeholders throughout the participatory modeling processes. The team lead (J.B.), who previously acted as the project champion and physician lead in earlier participatory modeling efforts [34], was the senior medical information officer for the health authority when this study was conducted. She played a key role in advocating for modeling approaches and interpreting modeling concepts and results in languages friendly to the stakeholders to facilitate knowledge translation. The team was in direct and frequent communication with decision-makers and public health officials.
3. *Problem conceptualization*: The primary objective of the modeling team was to support the decision-makers in addressing the urgent decision-making needs. It is worth noting that the team developed several COVID-19 transmission models, using different modeling approaches, to address different objectives and policy questions [2]. The hybrid COVID-19 model presented in this study focused on addressing questions related to non-pharmaceutical public health interventions and hospital capacity planning in the early pandemic: (1) How many infections and deaths are expected under different intervention scenarios and model assumptions? (2) What are the estimated acute care resource needs? (3) What are the effects of non-pharmaceutical public health interventions (e.g., contact tracing, physical distancing, quarantine, and isolation) on those outcomes?
4. *Model implementation, model validation, and model use*: The initial proof-of-concept hybrid model was continuously refined in response to policy requests posed by stakeholders. The model’s inputs and assumptions were reviewed and updated through a collaborative and iterative process. This included analyses of provincial epidemiological surveillance data, weekly scans of scientific evidence conducted by national and provincial research teams, and ongoing consultation with epidemiologists to update and review model inputs and results. This iterative process ensured that the hybrid model incorporated best estimates based on emerging evidence and new surveillance data. As the hybrid model was regularly adapted to reflect new evidence and evolving policy needs, we also conducted regular model validation through model verification, face validation, and cross-validation. Through routine meetings, the team leads presented model results in response to policy questions posed by decision-makers and medical health officers, while also addressing uncertainties and underlying model assumptions. The modeling results were also communicated to the general public through press briefings by the Saskatchewan Health Authority and the Ministry of Health on 28 April 2020 [38].
5. *Progress until June 2020*: The hybrid model presented in this study was actively used until June 2020, playing a key role in informing public health decisions during the early stages of the pandemic. As decision-making shifted towards planning for gradual reopening, addressing the subsequent waves, understanding new variants, and preparing for vaccination rollout at the end of the year, the focus of modeling efforts evolved. Other simulation models were developed by the modeling support team and were continuously used to inform public health policies throughout these transitions [2].

### 2.3. The Hybrid Simulation Model

The use of simulation models for studying the spread of emerging infectious diseases and designing interventions to inform the public health response has become increasingly important [1,10,39]. System Dynamics (SD) and agent-based modeling (ABM) are two commonly used simulation approaches to model the dynamics of disease spread, such as the spread of COVID-19 [1]. Although single methods like SD or ABM are effective for projecting the number of new cases, they often cannot help with operational challenges associated with healthcare systems such as acute care beds and resource needs. Using SD alone cannot adequately represent individual-level behaviors or individualized health interventions. Since each modeling method has its limitations, it is very rare for one single method to capture all aspects of the problems and provide support to decisions at various levels of complexity [39,40]. This highlights the need for hybrid simulation by combining different modeling approaches given its strong practical appeal to better represent the studied system and problem [39,40].

In this study, we developed and used a hybrid model of COVID-19 transmission to inform mitigation strategies and short-term hospital capacity planning in Saskatchewan, Canada. Our hybrid simulation approach combines SD, ABM, and discrete-event simulation (DES), leveraging strengths of each method. The hybrid model consists of three integrated sub-models: an age-structured SD stock and flow model to simulate COVID-19 transmission at the population level, an agent-based model to capture COVID-19 disease progression and conventional contact tracing at the individual level, and a DES to model COVID-19-related hospital patient flow. Our hybrid simulation approach ensures that both aggregate-level transmission dynamics (via SD) and individual-level contact tracing (via ABM) are represented, while DES allows for simulation of hospital patient flow, offering additional insights into resource utilization, such as acute care beds and intensive care beds. Our hybrid approach met the objectives of understanding COVID-19 transmission, evaluating mitigation measures, and planning for acute care capacity.

**Figure 1:**
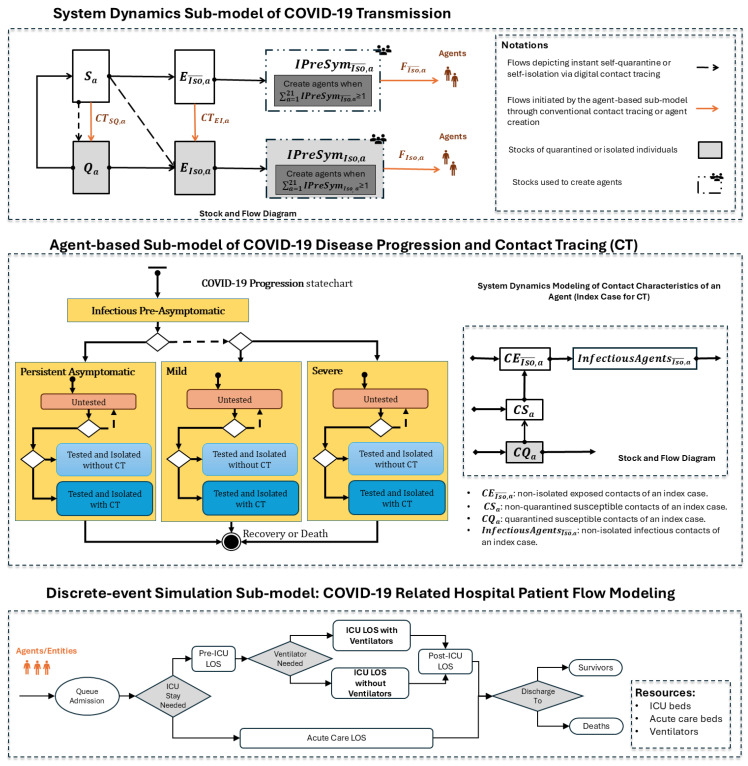
Hybrid model structure for simulating COVID-19 transmission, disease progression, and COVID-19-related hospital patient flow.

Figure 1 shows the model structure of the hybrid model. The following sections provide a detailed description of each sub-model in the hybrid model. Model parameters were derived from published studies or analyses of provincial epidemiological surveillance data (as seen in Table 1). The hybrid model was developed using AnyLogic 8.8.4 Professional Edition [41]. Although the hybrid model presented in this paper is not identical to the one used in March 2020, it closely resembles it and is a more recent version of the model. Each sub-model is described in detail in the following sections.

#### 2.3.1. System Dynamics Sub-Model of COVID-19 Transmission

This SD sub-model simulates COVID-19 transmission in the Saskatchewan population. The stock and flow diagram of this sub-model is illustrated in Figure 1. In the stock and flow diagram, rectangles represent “stocks”, which depict disease state and isolation status. Arrows denote “flows”, which characterize transitions between these stocks over time throughout the COVID-19 transmission process. The following stocks are used in the model, with each stratified into 21 distinct 5-year age groups *a*:

- Susceptible Individuals ($S_{a}$): individuals who are susceptible to COVID-19 infection and not in quarantine.
- Quarantined Susceptible Individuals ($Q_{a}$): susceptible individuals who are quarantined.
- Non-isolated Exposed Individuals ($E_{\bar{\mathrm{Iso}},a}$): latently infected individuals who are not isolated.
- Isolated Exposed Individuals ($E_{\mathrm{Iso},a}$): latently infected individuals who are isolated.
- Non-isolated Infectious Presymptomatic Individuals ($IPreSym_{\bar{\mathrm{Iso}},a}$): non-isolated infectious individuals who have not yet shown any symptoms of COVID-19 infection.
- Isolated Infectious Presymptomatic Individuals ($IPreSym_{\mathrm{Iso},a}$): infectious presymptomatic individuals who are isolated.

The stratification into 21 distinct 5-year age groups aligns with aggregate population data provided by Statistics Canada and the Saskatchewan Ministry of Health. Age group a=1 represents individuals aged 0–4 years, a=2 represents those aged 5–9 years, and so forth, with a=21 representing individuals aged 100 years and older (as detailed in Table A1 in Appendix A).

We include age-specific mixing patterns for the Saskatchewan population by introducing preferential interactions between different age groups [49]. Let $P_{\alpha ,a}$ denote the relative case-contact mixing preference between age group α and *a*. We define the mixing matrix $M_{\alpha ,a}$ as:

(1) $$M_{\alpha ,a}=\frac{P_{\alpha ,a}\frac{N_{a}}{N}}{\sum_{a=1}^{21}P_{\alpha ,a}\frac{N_{a}}{N}},$$

where *N* represents the total population, and $N_{a}$ represents the population size of age group *a*. $M_{\alpha ,a}$ is population-distribution-weighted, and indicates the fraction of contacts that an infectious case from age group α has with individuals from age group *a*. When $P_{\alpha ,a}=1$, we get random mixing with $M_{\alpha ,a}=\frac{N_{a}}{N}$. $M_{\alpha ,a}$ is normalized such that $\sum_{a=1}^{21}M_{\alpha ,a}=1$ for all α, reflecting the complete probability distribution of contacts for a case.

We define a set of differential equations to represent the transmission dynamics. Equation (2) captures the change in the number of susceptible individuals ($S_{a}$) in age group *a* over time:

(2) $$\frac{dS_{a}}{dt}=-\beta C\sigma \left(\sum_{\alpha =1}^{21}M_{\alpha ,a}I_{\alpha }\right)\frac{S_{a}}{N_{a}}-\left(1-\beta \right)C\sigma \left(\sum_{\alpha =1}^{21}M_{\alpha ,a}I_{\alpha }\right)\frac{S_{a}}{N_{a}}\rho +\frac{Q_{a}}{\gamma }-CT_{SQ,a}.$$

Susceptible individuals exit this stock via flows if they are infected, quarantined, or contact-traced (also seen in Figure 1). Quarantined susceptible individuals ($Q_{a}$) return to $S_{a}$ stock after quarantine. β is the probability of transmission per discordant contact—the probability of infection given contact between a susceptible individual and an infectious individual. It is a derived value in the model, based on $R_{0}$, *C* (contacts per case per day), and the duration of infectiousness parameters. $I_{\alpha }$ is the total number of *effective* infectious individuals in age group α. The term *effective* refers to the adjustment on the total number of infectious individuals to account for the reduced capacity to transmit the disease due to isolation or diagnosis (via testing), compared to those who remain unisolated and untested. The physical distancing level parameter (σ) ranges from 0 to 1, where σ=0 indicates complete physical distancing (no contact), and σ=1 represents the same level of physical distancing as that in pre-pandemic.

Equation (3) captures how the quarantined susceptible individuals in $Q_{a}$ stock changes over time. Individuals leave the stock when they complete their quarantine or when they are infected. At the same time, susceptible individuals enter the $Q_{a}$ stock if they are contact-traced or quarantined:

(3) $$\frac{dQ_{a}}{dt}=\left(1-\beta \right)C\sigma \left(\sum_{\alpha =1}^{21}M_{\alpha ,a}I_{\alpha }\right)\frac{S_{a}}{N_{a}}\rho -\beta C\sigma \left(1-\theta \right)\left(\sum_{\alpha =1}^{21}M_{\alpha ,a}I_{\alpha }\right)\frac{Q_{a}}{N_{a}}-\frac{Q_{a}}{\gamma }+CT_{SQ,a}.$$

Equations (4) and (5) track the changes of the number of infected individuals who are in the latent period over time:

(4) $$\frac{dE_{\bar{\mathrm{Iso}},a}}{dt}=\beta C\sigma \left(\sum_{\alpha =1}^{21}M_{\alpha ,a}I_{\alpha }\right)\frac{S_{a}}{N_{a}}\left(1-\rho \right)-\frac{E_{\bar{\mathrm{Iso}},a}}{l}-CT_{EI,a},$$

(5) $$\frac{dE_{\mathrm{Iso},a}}{dt}=\beta C\sigma \left(\sum_{\alpha =1}^{21}M_{\alpha ,a}I_{\alpha }\right)\frac{S_{a}}{N_{a}}\rho +\beta C\sigma \left(1-\theta \right)\left(\sum_{\alpha =1}^{21}M_{\alpha ,a}I_{\alpha }\right)\frac{Q_{a}}{N_{a}}-\frac{dE_{\mathrm{Iso},a}}{l}+CT_{EI,a}.$$

Equations (6) and (7) model the changes in the stocks of pre-symptomatic infectious individuals. Infected individuals move to these two stocks after completing the latent period:

(6) $$\frac{dIPreSym_{\bar{\mathrm{Iso}},a}}{dt}=\frac{E_{\bar{\mathrm{Iso}},a}}{l}-F_{\bar{\mathrm{Iso}},a},$$

(7) $$\frac{dIPreSym_{\mathrm{Iso},a}}{dt}=\frac{dE_{\mathrm{Iso},a}}{l}-F_{Iso,a}.$$

The interaction between the SD sub-model and the ABM sub-model occurs through two primary mechanisms. The first involves the flows in Figure 1 labeled $CT_{SQ,a}$ and $CT_{EI,a}$, which are triggered by conventional contact tracing in the ABM sub-model. The second mechanism dynamically generates agents based on the stock values of $IPreSym_{\bar{\mathrm{Iso}},a}$ and $IPreSym_{\mathrm{Iso},a}$. Agents are created when the respective flows accumulate enough individuals to constitute an entire agent; that is, when $\sum_{a=1}^{21}IPreSym_{\bar{\mathrm{Iso}},a}\geq 1$ or $\sum_{a=1}^{21}IPreSym_{\mathrm{Iso},a}\geq 1$. Following the creation of an agent in a specific age group, the corresponding stock for that age group decreases by 1, reflecting that this quantity is now accounted for as a specific agent. This process continues until the stock value in any age group drops to less than 1. If the total stock value across all age groups remains above 1 after the initial round of agent creation, additional agents are generated, where the age group associated with a given agent is drawn from a custom distribution based on $IPreSym_{\bar{\mathrm{Iso}},a}$ or $IPreSym_{\mathrm{Iso},a}$. The creation of agents continues until $\sum_{a=1}^{21}IPreSym_{\bar{\mathrm{Iso}},a}<1$ or $\sum_{a=1}^{21}IPreSym_{\mathrm{Iso},a}<1$.

#### 2.3.2. Agent-Based Sub-Model of COVID-19 Disease Progression and Contact Tracing

The agent-based sub-model was structured to capture disease progression and contact tracing at the individual level (seen in Figure 1). Through interaction with the SD sub-model, individuals are instantiated as agents once they become infectious, with the potential to spread the COVID-19 infection. We keep track of the isolation status of each agent. Each agent has a “COVID-19 Progression” statechart, which characterizes the state of that individual with respect to the natural history of COVID-19 infection. The agent starts in the “*Infectious Pre-Asymptomatic*” state in the statechart and then enters into one of three mutually exclusive composite states based on disease severity: “*Persistent Asymptomatic*”—individuals who remain asymptomatic throughout the course of infection, “*Mild*” – individuals with mild symptoms that do not require hospital care, and “*Severe*”—individuals with severe symptoms that require hospitalization. Within each of the three composite states—higher-level states that contain other substates—we modeled the test-isolate-trace-quarantine intervention. After entering one of the composite states (e.g., “*Mild*”), the initial inner state is “*Untested*”. The agent may stay “*Untested*” throughout the course of its infection without being diagnosed, or the agent may undergo testing and isolation ending in either the “*Tested and Isolated without CT*” state, or the “*Tested and Isolated with CT*” state, depending on whether that individual has been contact-traced. In the end, the agent transitions into the “*Recovery or Death*” final state. In line with the evidence available at the time, we assumed that recovered individuals remained immune and would not be re-infected in the 1-year simulation period.

Each agent also has an age-structured System Dynamics sub-model tracking the time course of contacts for an individual in its age group at the aggregate level. This component is a deterministic stock-and-flow model simulating how a single agent in a given age group transmits the infection to its contacts during its infectious period. These contacts could also be infected by other infectious agents in the population during the same period. This component continuously tracks the following types of contacts for an infectious agent via stocks: (1) exposed contacts or infectious contacts who are not isolated (represented by the stocks $CE_{\bar{\mathrm{Iso}},a}$ and $InfectiousAgents_{\bar{\mathrm{Iso}},a}$), allowing for isolation through conventional contact tracing, and (2) susceptible contacts who have interacted with the infectious agent but are neither infected nor quarantined (represented by the stock $CS_{a}$), who can potentially be placed in quarantine via contact tracing. We used the same parameters in the SD sub-model to simulate the transmission of an infectious agent.

Depending on the contact tracing probability, test delay, and the fraction of contacts traced, when an agent is contact-traced at a given time *t*, a proportion of the quantity in the stock $CE_{\bar{\mathrm{Iso}},a}$ is instantaneously moved from $E_{\bar{\mathrm{Iso}},a}$ to $E_{\mathrm{Iso},a}$ via the flows $CT_{EI,a}$ in the SD sub-model of COVID-19 transmission. Simultaneously, a proportion of the quantity in $CS_{a}$ is moved from the $S_{a}$ stock to the $Q_{a}$ stock via the flow $CT_{SQ,a}$. These two transitions serve to isolate individuals identified through conventional contact tracing. Additionally, a proportion of individuals in the $InfectiousAgents_{\bar{\mathrm{Iso}},a}$ stock are also isolated by randomly selecting unisolated infectious agents in the ABM sub-model and changing their status to “isolated”.

#### 2.3.3. Discrete-Event Simulation Sub-Model for COVID-19 Related Hospital Care

The DES sub-model simulates the flow of severe COVID-19 cases through a generic hospital system. It interacts with the “COVID-19 progression” statechart in the ABM sub-model; agents in the “*Severe*” composite state, following a delay for care-seeking, are injected into this DES sub-model as entities requiring hospital care. Severe COVID-19 patients first enter a queue for acute care admission. A decision node then determines whether the inpatient requires admission to the intensive care unit (ICU). The hospital length of stay (LOS) for patients requiring intensive care is divided into three parts: pre-ICU LOS, ICU LOS, and post-ICU LOS. For ICU LOS, the model distinguishes between patients who need ventilators and those who do not. The DES sub-model includes three types of resources: acute care beds, ICU beds, and ventilators. These resources are assumed to be unlimited; however, their utilization is analyzed to assess acute care resource needs and inform healthcare resource planning for COVID-19.

### 2.4. Model Validation

We conducted various types of model validation. This included verification, face validation, external validation, and cross validation [66]. For model verification, we regularly examined the equations and implementation in code through peer review. Despite strong interest in the COVID-19 models after the initial presentation given to the provincial leadership team in mid-March 2020, a set of questions were raised regarding model transparency and validity. To address these questions, we conducted face validation with epidemiologists, analysts, researchers, and modelers. For example, on 28 March 2020, we held a model challenge session to review the model structure, parameter values, and assumptions with regional leaders, epidemiologists and stakeholders who were interested in the model details. A variety of questions were raised for clarification and discussion during this meeting, including but limited to: (1) whether the sources of evidence used for model inputs were the most recent and applicable to the Saskatchewan population; (2) how asymptomatic cases were represented in the model; (3) whether the model accounted for limited testing capacity; and (4) how significantly the number of contacts per case per day impacted the model’s outcomes and contact tracing. In response to the questions raised, changes were made to the model structure, and sensitivity analyses were conducted. Face validation helped improve the model, enhance its credibility with experts, and increase the acceptance of model results. We also communicated with experts and stakeholders transparently about the uncertainties and unknowns.

The basic reproduction number ($R_{0}$) is an important parameter for projecting the epidemic trend, and it also had great uncertainty in the early pandemic and could vary across regions and countries due to difference in population density and social behaviors [67]. To address this, we reviewed $R_{0}$ estimates from the literature and independently estimated $R_{0}$ for Saskatchewan using maximum likelihood estimation method based on 1-month COVID-19 case data [68,69]. In addition to estimating $R_{0}$, we further calibrated this parameter within the model by comparing the model projections against the real COVID-19 data in Saskatchewan from 12 March to 12 April 2020. We used the Mean Squared Error (MSE) metric for assessing the goodness-of-fit of the model. MSE quantifies the average squared difference between the observed data and the model’s simulated values, with lower values indicating a closer fit: $\mathrm{MSE}=\frac{1}{n}\sum_{i=1}^{n}$(real_data_*i*_ − simulated_data_*i*_)^2^, where *n* represents the total number of data points. By cross-validating estimates of $R_{0}$ from various sources and using different methods, we were able to gain confidence in its uncertainty ranges, which enabled us to conduct sensitivity analyses on $R_{0}$ with greater rigor.

We also conducted cross validation—“examining different models that address the same problem and comparing their results” [66], based on requests from stakeholders. The modeling team had developed several COVID-19 transmission models in the early pandemic, we also received modeling results from models developed by Public Health Agency of Canada (PHAC). We examined the differences among the results from various models and their causes in terms of assumption and model inputs. Undertaking this process not only helped us gain insights into such differences but also built trust in our hybrid model with the stakeholders.

### 2.5. Intervention Scenarios

We considered the following interventions to mitigate the spread of infection: physical distancing, case detection and isolation, conventional contact tracing with quarantine, and digital contact tracing with quarantine. Physical distancing reduces the contact rate per day (*C*) for the entire population. Case detection and isolation work together to reduce the effective transmissibility of the confirmed cases, with testing serving as the primary method for detecting (or diagnosing) cases. Conventional contact tracing builds on the case detection and isolation intervention: once an infected individual is diagnosed and isolated, their contacts who may have been exposed are identified and placed in quarantine (or isolation if contacts are infectious). Quarantine was assumed to reduce the risk of infection. Isolation alone (without testing) was assumed to slightly reduce the infectivity of infectious individuals. We also accounted for the delay associated with testing and contact tracing, from symptom onset to case isolation and contact tracing. This delay reflects the time taken by individuals to seek care and the speed at which they are tested and traced. We also considered digital contact tracing as a “what-if” intervention in response to stakeholders’ interest. Especially with the prevailing use of mobile phones, digital contact tracing could potentially be done through a mobile phone app, allowing contacts to be notified instantly and placed in quarantine. Details of the parameter configurations for each intervention are presented in Table 2. Some characteristics of those parameter assumptions bear note. In the baseline scenario, we assumed a degree of physical distancing, testing and case isolation, and conventional contact tracing. The baseline value for “contacts instantly quarantined or isolated (ρ)” does not represent digital contact tracing but instead reflects the mandatory self-quarantine policy implemented for international travelers and their contacts in March 2020 in Saskatchewan [51]. Travel-related cases were a primary source of infection in the early pandemic, so we assumed that a proportion of potentially exposed individuals (ρ) were immediately self-quarantined or self-isolated in compliance with public health orders. For the conventional contact tracing intervention, we varied the use of mass testing or faster testing with contact tracing, adjusting the proportion of cases being traced from 30% in the baseline to 90% in 30% intervals. “Contacts traced with instant quarantine or isolation (%)” is the fraction of contacts successfully traced and immediately quarantined for a confirmed case. We set its baseline value at 60%. For digital contact tracing, we assumed that a higher proportion of contacts—ranging from 40% to 60%—are instantly traced and quarantined upon exposure.

We initiated the transmission in our hybrid model using daily data on travel-related COVID-19 cases in Saskatchewan, collected from 12 March to 12 April 2020. There are 128 travel-related COVID-19 cases in this period. As various travel restrictions were implemented in the early pandemic, we assumed that the number of travel-related COVID-19 cases would drop to zero after 12 April 2020. We ran 30 iterations of each simulated scenario, with a model run time of 10 min per iteration. We ran the model for 1 year starting from 2 March 2020. The simulation runs were executed on a Lenovo ThinkStation P330 with Intel® Core™ i7-9700T processor (Intel Corporation, Santa Clara, CA, USA) and 64 GB of memory under Windows 11 Pro (Version 23H2).

## 3. Results

### 3.1. Model Validation

**Figure 2:**
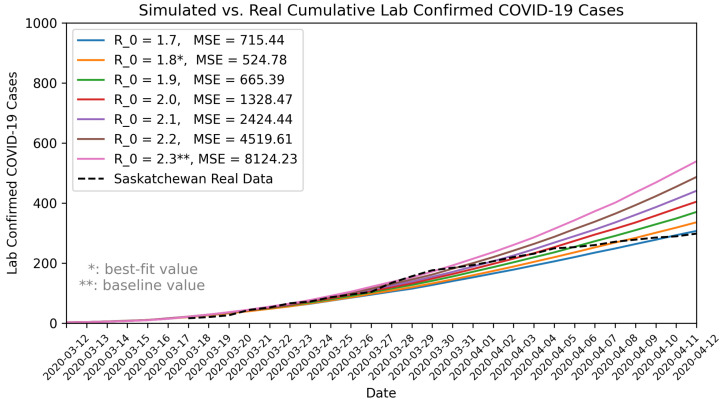
Comparison of model outputs with observed COVID-19 data in Saskatchewan. MSE: Mean Squared Error.

By varying the basic reproduction number ($R_{0}$) between 1.75 and 2.6, we found that an $R_{0}$ of 1.8 best matched the observed COVID-19 data for the 1-month validation period (12 March to 12 April 2020). Using the maximum likelihood estimation method [68,69], we independently estimated $R_{0}$ for Saskatchewan to be 1.84, with a 95% confidence interval of 1.46 to 2.3. The calibration result ($R_{0}=1.8$) and the independently estimated value ($R_{0}=1.84$) using a different approach closely aligned. We presented the simulated results for different $R_{0}$ compared to the observed COVID-19 data in Figure 2.

We communicated our estimates and uncertainty range of $R_{0}$ with stakeholders and domain experts. In response to the stakeholder needs and for the purpose of pandemic preparedness, particularly contingency planning for a “worst-case” scenario, we used a $R_{0}$ of 2.3 for our baseline scenario (also seen in Table 1). This value, representing the upper bound of the 95% confidence interval, reflects a more conservative estimate that aligns with stakeholders’ interest for contingency planning. In addition, given the limited testing capacity in the early pandemic, the actual COVID-19 cases might have been under-reported or subject to reporting delays, potential leading to underestimates of the true $R_{0}$. Furthermore, a number of mitigation measures were implemented during this 1-month period, which also could contribute to an underestimated $R_{0}$. Therefore, using a higher $R_{0}$ ensures that preparedness efforts are adequate, even if the actual epidemic trend is less severe than the worst-case assumption. In pandemic preparedness, the goal is to plan for the worst case, but not predicting the most likely scenario.

For cross-validation, we compared our 1-year projections with those from an independently developed COVID-19 model by the PHAC. The PHAC model projected an infection rate of 25% to 50% and 3000 to 6000 deaths for Saskatchewan under a “flatten the curve” scenario that included social distancing, case detection, contact tracing, and quarantine. In comparison, our baseline scenario with $R_{0}=2.3$ projected a 30.7% infection rate (371,809 cases) and 3271 deaths, falling within PHAC’s range but toward the lower end.

### 3.2. Simulation Results

In the early COVID-19 pandemic, the risk of mortality and the potential collapse of the acute care system were two major concerns for decision-makers in Saskatchewan, Canada. To address these concerns and help flatten the epidemic curve, we collaborated with decision-makers through participatory modeling processes to evaluate a set of mixed intervention scenarios, which could be implemented quickly. We explored numerous mixed scenarios and present our key findings here. The key model outcomes include the total number of true infections (the total number of infected individuals), cumulative confirmed COVID-19 cases, and cumulative deaths over a 1-year period, as shown in Table 3.

**Figure A1:**
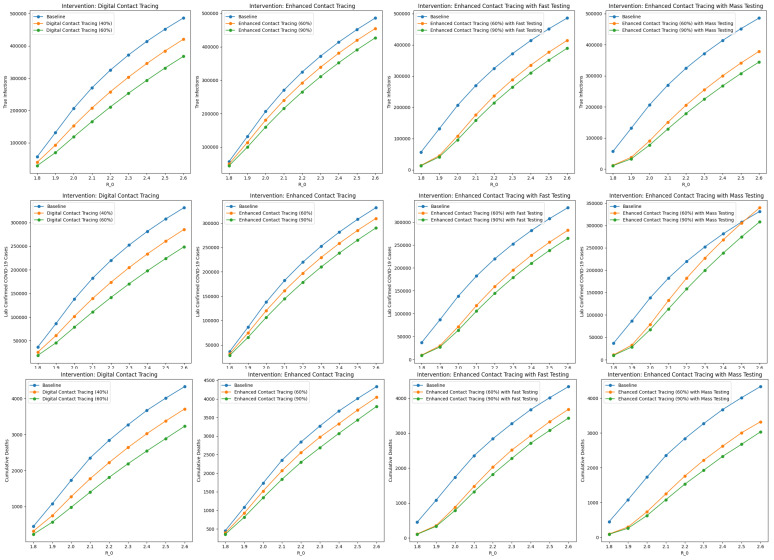
Sensitivity analyses on the basic reproduction number ($R_{0}$).

Among the intervention scenarios with 60% contact tracing, enhanced contact tracing with mass testing was most effective in reducing both true infections and cumulative deaths, and compared favorably to enhanced contact tracing with faster testing. Although faster testing allows for quicker case isolation and contact tracing, mass testing with contact tracing has a greater overall impact on reducing transmission and mortality. In addition, although the number of confirmed COVID-19 cases is higher with mass testing compared to faster testing, this should be interpreted cautiously. The higher number of confirmed COVID-19 cases reflects improved case detection and isolation, and should not be interpreted as indicative of a worsened underlying epidemiological situation. By combining contact tracing with mass testing, we can monitor the COVID-19 spread in the population more accurately. Similar trends were observed for scenarios with 90% contact tracing. Sensitivity analyses on $R_{0}$ are presented in Figure A1 in Appendix A.

**Figure 3:**
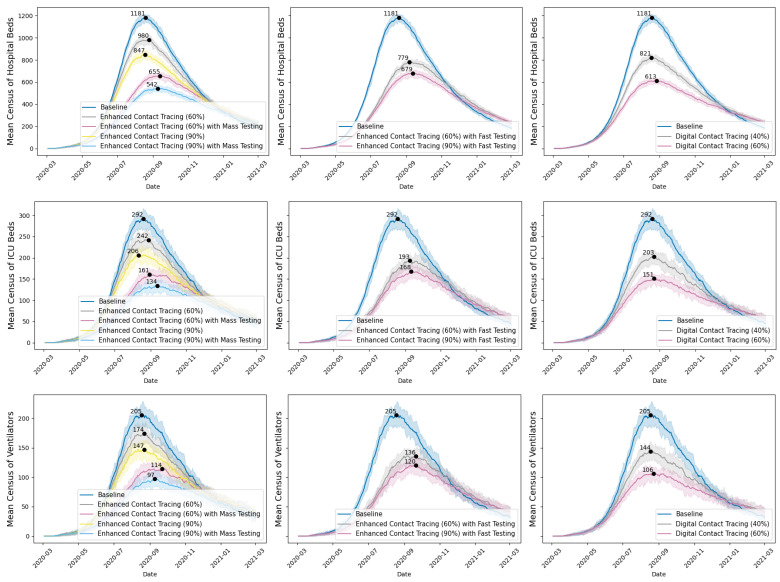
Mean census of hospital beds, ICU beds, and ventilators for COVID-19 inpatients under various scenarios, with peak values and 10th and 90th percentile ranges.

Figure 3 reports the mean census of hospital beds, ICU beds, and ventilators (with 10th to 90th percentiles across 30 iterations) per simulated scenario over the one-year period. Enhanced contact tracing could significantly lower the peak mean census of acute care resources, with the peak census occurring in August and September 2020. Our focus on the census of hospital beds, ICU beds, and ventilators was guided by stakeholders’ inputs and interests. As acute care resources are scarce, sudden surges in demand for these resources could strain the healthcare system, resulting in higher mortality rates due to insufficient capacity. The peak census and its timing represent the worst situation for resource demand. Understanding and mitigating the peak census allows for better preparation to handle the highest anticipated demand for acute care resources, thereby preventing the crisis.

Digital contact tracing has been deployed via apps in several countries, such as China, Australia, and UK [70]. Provincial public health professionals were also interested in its potential impact in Saskatchewan. We simulated two hypothetical scenarios to showcase the impact of extremely rapid contact tracing on COVID-19 transmission, assuming a proportion of contacts are immediately notified and isolated upon COVID-19 exposure. This differs from conventional contact tracing, in which the COVID-19 cases need to be confirmed before contact tracing can start. With digital contact tracing, we saw a significant drop in true infections and cumulative deaths. However, with regard to digital contact tracing, strong ethical concerns were raised about data privacy and protection, voluntary adoption versus mandatory use, the uptake of such apps in the Saskatchewan population, and the accuracy of the sensors in detecting contacts.

### 3.3. Policy Responses

This section describes the outcomes of using the participatory modeling processes and the hybrid model—highlighting how the model informed mitigation measures implemented in Saskatchewan and how stakeholders used the model results.

Within 1 week of the first COVID-19 cases being confirmed in Saskatchewan, the leadership team and executive leadership team from the provincial health authority had reviewed the preliminary modeling results of various intervention scenarios, and this included simulation results of contact tracing using an earlier version of the hybrid model presented in this paper. On 26 March 2020, the modeling team presented these modeling results—including contact tracing scenarios—to the Saskatchewan Chief Medical Health Officer, stakeholders, and public health professionals. We highlighted the effectiveness of enhanced contact tracing with mass testing in mitigating the COVID-19 spread if implemented early enough in Saskatchewan. We also noted that Singapore and South Korea had achieved some early success through mass testing and contact tracing at that time. On 28 March 2020, the Government of Saskatchewan released the “Testing and Contact Tracing Plan” to the public,

“*in addition to ongoing testing, the Ministry of Health will deploy additional staff to the Saskatchewan Health Authority (SHA) to assist with contact tracing. This initiative is aimed at critical identification to better ensure cases are detected and followed up on in a timely manner, and to help prevent further transmission of COVID-19*” [71,72].

On March 30, 2020, “*the Saskatchewan Health Authority (SHA) already has up to 150 people involved in contact tracing. Over the coming days, 50 additional staff from across government will be moved into this key role. The need for additional resources will be assessed as the situation evolves.... Testing locations will continue to be expanded as demand warrants* [73].

By the end of March 2020, the province had significantly ramped up testing, ranking third in Canada for travel and non-travel-related testing [71].

Saskatchewan Health Authority also released preliminary COVID-19 modeling results to the public on 8 April 2020, reporting on a range of potential outcomes based on assumptions, public policies, and compliance. These results were from another COVID-19 transmission model developed by the modeling support team. Later, on 28 April 2020, updated health system planning was released to the public. This update included a revised acute care surge planning scenario, which was based on simulation results from the hybrid model presented in this study, using an earlier version of the model with different assumptions. The revised acute care planning scenario suggested a significant reduction in COVID-19 transmission, signaling that Saskatchewan was in a better position to manage the pandemic as a result of the interventions taken [38]. The province continued its efforts to expand the testing capacity and speed up contact tracing [74].

## 4. Discussion

In contrast to many academic COVID-19 models developed during the pandemic in hopes of attracting attention and adoption from health systems, we collaborated directly with stakeholders and public health professionals within the healthcare system through participatory modeling processes to develop and use a hybrid simulation model of COVID-19 transmission and undertake analyses that were tailored to the local context to support rapid decision-making in the early COVID-19 pandemic in Saskatchewan, Canada.

Although dynamic modeling and simulation techniques have been used as a systematic approach to understand the dynamics of infectious diseases and other healthcare challenges for decades, their adoption as central tools for real-world decision-making remains limited [11,75,76,77,78,79]. Lack of stakeholder engagement in the life-cycle of a simulation study is an important contributing factor to the low-level implementation of simulation results in healthcare settings [12,80,81]. Participatory modeling has been proposed as a way to engage stakeholders in the modeling process, supporting shared learning or the decision-making processes [15,17,18,25,82,83]. In this simulation study, we incorporated participatory modeling processes, drawing on experience and lessons from an earlier participatory approach used within the same healthcare system [34]. This close partnership with the stakeholders ensured that the hybrid model was inherently relevant for decision-making. We believe that several key factors contributed to the successful adoption of the hybrid model’s simulation results in informing rapid decision-making: (1) the high priority of addressing the COVID-19 pandemic given its unprecedented scale; (2) rapid response of the modeling team; (3) trust and experience gained from the earlier participatory modeling efforts, which facilitated buy-in for the modeling approach, expertise of the modelers, and the modeling results; (4) joint development of the hybrid model to meet the evolving policy needs; (5) constant review and updating of model assumptions and key model inputs as new evidence emerged; and (6) regular model validation to ensure the model’s credibility.

Effective contact tracing is a crucial public health measure to mitigate the spread of infectious diseases [84,85,86], and it has been employed in many countries since the beginning of the COVID-19 pandemic [87,88,89]. A lot of research has examined the effectiveness of contact tracing in the context of COVID-19 pandemic [90,91,92]. Our findings on the effectiveness of enhanced contact tracing with mass testing align with other studies, which suggest that intensive contact tracing with mass testing is more effective in controlling COVID-19 spread than the test and trace method, and even more effective if combined with social distancing measures [91,93,94].

Also bearing emphasis is the novelty of the hybrid simulation approach used in this study, which combines SD, DES, and ABM. The use of a hybrid simulation approach helps overcome the limitations of using a single simulation technique [40,95]. We chose SD for quickly modeling COVID-19 transmission at the aggregate level, as using ABM for infectious disease modeling often face challenges such as lack of data (e.g., social networks, contact graph, mobility, risk and protective behavior, and individual characteristics), greater model complexity, and computational burden. While modeling contact tracing is best conducted at the individual-level, given the individual-based character of the contact tracing process and the need to track the health status of single individuals and the time course of contacts [85,96,97]. We used ABM to model contact tracing, which is usually the first choice to formulate the contact tracing process in a simulation model. SD is not ideal for modeling contact tracing, as the contact tracing process cannot be directly formulated given the aggregate nature of the SD approach [97]. For hospital patient flow, we chose DES, which allows us to model patient flow as a system of queues and workflows composed of stochastic processes with dependency on acute care resources that may be constrained. This was particularly relevant as hospital capacity planning was one primary concern in the early pandemic. Using a hybrid simulation approach, we leveraged the advantages of multiple simulation methods to address more complex issues and better capture the real-world scenarios.

This study also has some limitations. We made various assumptions in the model. In the early pandemic, it was particularly challenging to estimate the COVID-19 transmission accurately due to uncertainty surrounding key model inputs such as the basic reproduction number, proportion of persistent asymptomatic cases, and assumptions regarding human risk and protective behaviors. Additionally, although we stratified the population by age group and incorporated age-specific mixing patterns, within this work, we did not account for location-specific mixing patterns (rural versus urban). We also did not consider false positives in testing, which may have unintended implications for the mass testing intervention. Furthermore, the model did not account for waning of immunity. We communicated clearly with stakeholders about the model assumptions, key model inputs used, and model limitations when presenting model results. This transparency was vital in maintaining the credibility of the hybrid model in the face of a high degree of uncertainty in the early pandemic. For future work, the model could be further expanded by enhancing the patient flow component to capture service-level patient flow by hospital.

## 5. Conclusions

This study successfully engaged a broad range of stakeholders and public health officials through participatory modeling processes in the early COVID-19 pandemic in Saskatchewan, Canada. Using a hybrid model of COVID-19 transmission tailored to the policy needs of the stakeholders, we directly informed and helped validate the public health interventions implemented by the provincial government. Enhanced contact tracing with mass testing was shown to significantly reduce COVID-19 transmission, mortality, and the census of hospital and ICU beds.

## Figures and Tables

**Table 1:** Model parameters and baseline values used in the hybrid simulation model.

Parameter Name	Baseline Value	Plausible Range	Source ^a^
*Parameters used in System Dynamics Sub-model of COVID-19 Transmission *			
Mean latent period (*l*), day	3.7	(2–5.5)	[42,43,44,45]
Basic reproduction number ($R_{0}$)	2.3	(1.8–3.2)	[43,46,47]
Contacts per case per day (*C*)	10	(5–19)	[48,49,50]
Average duration of quarantine (γ), day	14	(10–14)	[51]
Reduction in exposure risk due to quarantine (θ)	0.64	(0.64–0.9)	[52]
Reduction in infectivity for diagnosed isolated cases	0.64	(0.64–0.9)	[52]
Reduction in infectivity for undiagnosed isolated symptomatic individuals	0.1	(0.05–0.1)	Assumed
Reduction in infectivity for undiagnosed isolated asymptomatic individuals	0.05	(0.05–0.1)	Assumed
Proportion of contacts instantly quarantined or isolated (ρ)	0.2 ^b^	(0.1–0.3)	Assumed
Physical distancing level relative to pre-pandemic level (σ)	0.75	(0.6–0.95)	[53]
*Parameters used in Agent-based Sub-model of COVID-19 Disease Progression*			
Duration of infectiousness before symptom onset, day	2	(1–2.9)	[45,54,55]
Duration of infectiousness for mild or asymptomatic cases after pre-asym, day	6.8	(4–9.5)	[43,56]
Duration of infectiousness from symptom onset for severe cases, day	5.9	(3.2–7)	[43,54,56]
Duration from symptom onset to hospital admission for severe cases, day	4	(3–12)	[57,58]
Proportion of pre-symptomatic individuals who never develop symptoms	0.3	(0.17–0.4)	[59,60,61]
*Parameters used in Discrete-event Simulation Sub-model of COVID-19 Related Hospital Patient Flow*			
Average length of stay for non-ICU inpatients ^c^, day	8	(4.1–14)	[62,63]
Average pre-ICU length of stay for ICU inpatients, day	3	(0–3)	[57,62]
Average ICU length of stay for ICU inpatients ^c^, day	8	(4–12)	[57,62]
Average post-ICU length of stay for ICU inpatients, day	3	(0–3)	[57,62]
Case fatality rate—ICU inpatients	0.49	(0.22–0.5)	[64]
Case fatality rate—non-ICU inpatients	0.05	(0.05–0.1)	[57,63]
Proportion of ICU inpatients requiring ventilation	0.71	(0.42–0.71)	[62]
Proportion of symptomatic cases requiring hospitalization by age group	– ^d^	(0.01–0.27)	[65]
Proportion of hospitalized cases requiring ICU admission by age group	– ^d^	(0.05–0.71)	[65]

ICU: intensive care units. ^a^ Reviewed and updated on a weekly basis based on ongoing scans of new evidence and epidemiological data. ^b^ The baseline value mimics the mandatory self-quarantine policy implemented for international travelers and their contacts in Saskatchewan in March 2020 in Saskatchewan. ^c^ Lognormal distributions were fitted and used for length of stay. ^d^ Seen in Table A1 in Appendix A.

**Table 2:** Configured parameters for simulated intervention scenarios.

Category	Target Population	Configured Parameters	Simulated Intervention Scenarios ^1^				
			**Baseline**	**Enhanced CT**	**Enhanced CT with Fast Testing**	**Enhanced CT with Mass Testing**	**Digital CT**
**Physical** **Distancing**	General population	Physical distancing start date	3/18/2020				
		Physical distancing level relative to pre-pandemic (σ), %	75				
**Case** **Detection,** **Isolation,** **and** **Contact** **Tracing**	Persistent asymptomatic cases	Time to test/isolation post-incubation, day	4		3		
		Case tested and isolated, %	60			90	
		Confirmed cases traced, %	30	60, 90	60, 90	60, 90	
		Contacts traced with instant quarantine or isolation, %	60				
	Mild cases	Time to test/isolation after symptom onset, day	3		2		
		Case tested and isolated, %	70			90	
		Confirmed cases traced, %	30	60, 90	60, 90	60, 90	
		Contacts traced with instant quarantine or isolation, %	60				
	Severe cases	Time to test/isolation after symptom onset, day	3		2		
		Case tested and isolated, %	100				
		Confirmed cases traced, %	30	60, 90	60, 90	60, 90	
		Contacts traced with instant quarantine or isolation, %	60				
	-	Conventional contact tracing start date	3/12/2020				
**Digital** **Contact Tracing**	Potentially exposed	Digital contact tracing start date	-				3/30/2020
		Contacts instantly quarantined or isolated (ρ), %	20				40, 60

CT: contact tracing. ^1^ Cells without values indicate that baseline values are used.

**Table 3:** COVID-19 Intervention Scenarios and Simulation Outcomes.

Simulated Intervention Scenario	Scenario Name and Configuration	True Infections ^1^	Confirmed COVID-19 Cases	Cumulative Deaths
Baseline	Baseline	371,809	252,504	3271
Enhanced Contact Tracing	Enhanced Contact Tracing (60%)	338,623	229,383	2969
	Enhanced Contact Tracing (90%)	310,612	209,926	2686
Enhanced Contact Tracing with Fast Testing	Enhanced Contact Tracing (60%) with Fast Testing	288,638	195,246	2515
	Enhanced Contact Tracing (90%) with Fast Testing	264,660	178,737	2275
Enhanced Contact Tracing with Mass Testing	Enhanced Contact Tracing (60%) with Mass Testing	255,134	227,211	2218
	Enhanced Contact Tracing (90%) with Mass Testing	224,838	199,751	1927
Digital Contact Tracing	Digital Contact Tracing (40%)	303,211	204,862	2641
	Digital Contact Tracing (60%)	253,343	170,485	2189

^1^ Saskatchewan has a population of 1.211 million.

## Data Availability

The hybrid model is available from the corresponding author (Y.T.) upon reasonable request.

## Appendix A

**Table A1:** Symptomatic cases requiring hospitalization and intensive care [65].

Age Group	Age Group ID	Symptomatic Cases Requiring Hospitalization, %	Hospitalized Cases Requiring Intensive Care, %
0 to 4 years	1	0.1	5.0
5 to 9 years	2	0.1	5.0
10 to 14 years	3	0.3	5.0
15 to 19 years	4	0.3	5.0
20 to 24 years	5	1.2	5.0
25 to 29 years	6	1.2	5.0
30 to 34 years	7	3.2	6.3
35 to 39 years	8	3.2	6.3
40 to 44 years	9	4.9	6.3
45 to 49 years	10	4.9	6.3
50 to 54 years	11	10.2	12.2
55 to 59 years	12	10.2	12.2
60 to 64 years	13	16.6	27.4
65 to 69 years	14	16.6	27.4
70 to 74 years	15	24.3	43.2
75 to 79 years	16	24.3	43.2
80 to 84 years	17	27.3	43.2
85 to 89 years	18	27.3	70.9
90 to 94 years	19	27.3	70.9
95 to 99 years	20	27.3	70.9
100 years and over	21	27.3	70.9
